# Supplementary material for: A prevalence of Arthropterygius (Ichthyosauria: Ophthalmosauridae) in the Late Jurassic—earliest Cretaceous of the Boreal Realm
Source: PeerJ. 2019 Apr 29;7:e6799. doi: 10.7717/peerj.6799 (PMC6497043; doi:10.7717/peerj.6799)
Supplement: Supplemental Information 1 — Five supplemental tables with measurements, 5 suplemental tables with data for PCA, eight suplemental figures, table with new characters for phylogenetic analysis, data on character coding revision and new scores. [file peerj-07-6799-s001.pdf]

Supplementary information for '*A prevalence of Arthropterygius* (Ichthyosauria: Ophthalmosauridae) in the Late Jurassic – earliest Cretaceous of the Boreal Realm'

by Nikolay G. Zverkov & Natalya E. Prilepskaya

Supplemental tables

Table S1. Selected measurements for ***Arthropterygius chrisorum***

**Quadrate**	**CCMGE 3-16/13328**	**CCMGE 17-44/13328**	**PMO 222.669 l/r**
Maximal length of ventral portion (condylar region)	53,7	64	83/81
Width of the condyle (acond)	23,5	23(compressed)	42.5/44.2
Height	84	102	124/118
Length in the region of quadrate foramen	44	49	60/-

**Basisphenoid**	**CCMGE 3-16/13328**	**CCMGE 17-44/13328**	**CNM 40608**	**PMO 222.669**
Length	57	63	78	73
Width	34x2=68	74 [est]	98	83
Height	32,8	42	~50	?

**Scapula**	**CCMGE 3-16/13328**	**PMO 222.655**	**SGM 1573**	**PMO 222.669**
Length	135,7	123	-	205/190
Width of proximal end	85,7	67	-	141/125
Width/thickness of the shaft	35,5/15,4	36/-	87/40	58/55

**Interclavicle SGM 1573**	
width	250
length	150

**Coracoid** (left/right)	**CCMGE 3-16/13328**	**PMO 222.669**
Anteroposterior length	141/145	260/265
Mediolateral width	130/130	210/215
Length of medial symphysis	95/94	170
Thickness of the medial facet	34/34	80
Length of glenoid contribution	64/58	77inc/96
Length of scapular facet	27/20	72/66

**Humerus**	**CCMGE 3-16/13328**	**CCMGE 17-44/13328**	**SGM 1573**	**PMO 222.669** (L/R)	**PMO 222.655**	**CNM 40608**
Proximodistal length	99	151	220	160/163	86	220
Width of proximal end	73	121	185	124/113.5	49[incompl.]	172
Height (thickness) of proximal end	27	60	95	62/63		97
Width of distal end (fU/fR/faae)	77.3(37/36/25)	122(50/45/39)	?(-/68/58)	121(52/49/40)//120(52/51/37)	60(34/30/25)	195(?)
Thickness of distal end (fU/fR/faae)	30(22/30/24)	47(38/45/38)	76(-/76/60)	54(41/54/45)//50(39.3/48.7/37)	30(20/30/25)	(?)
Width of the shaft	60	84	125	77/78	44	130

**Femur**	**CCMGE 17-44/13328**	**CNM 40608**
Proximodistal length	112	148
Width of proximal end	51	75
Width of distal end	55	86

Table S2. Measurements (mm) for vertebral centra of ***A. chrisorum*** **CCMGE 3-16/13328.**

**Field_No**	**hyp. No**	**L**	**H**	**W**	**H/L**	**H/W**
x	13	17,00	40	NA	2,35	
x	14	19,00	43	44	2,26	0,98
x	15	20,00	43	-	2,15	-
x	16	18,00	41,4	43,6	2,30	0,95
x	17	19,80	46	46	2,32	1,00
x	18	21,50	NA	47	-	-
x	19	23,60	NA	NA	-	-
x-8	20	24,30	NA	NA	-	-
x-7	21	22,30	NA	48,6	-	-
x-6	22	23,30	49,5	49,3	2,12	1,00
x-5	23	22,30	51	47,3	2,29	1,08
x-4	24	24,00	42	58,5	1,75	0,72
x-3	**25**	25,40	50	51	1,97	0,98
x-2	26	25,00	51	54	2,04	0,94
x-1	27	25,80	55	50	2,13	1,10
x1	28	24,00	52	47	2,17	1,11
x2	29	24,50	54	50	2,20	1,08
x3	30	24,70	57	56	2,31	1,02
x4	31	25,70	55,9	58,5	2,18	0,96
x5	32	25,00	62,4	60	2,50	1,04
x6	33	24,00	63	64	2,63	0,98
x7	34	24,00	61	64,4	2,54	0,95
x8	35	24,60	56	63	2,28	0,89
x9	36	24,30	56,4	61,3	2,32	0,92
x10	37	24,40	56	61	2,30	0,92
x11	38	24,20	62	60	2,56	1,03
x12	39	23,00	60	58	2,61	1,03
x13	40	22,70	NA	NA	-	-
x14	41	23,10	57,3	67	2,48	0,86
x15	42	23,00	58,8	68,7	2,56	0,86
x16	43	22,00	64,9	61,3	2,95	1,06
x17	44	20,10	60,8	62	3,02	0,98
x18	45	21,00	58,5	63,7	2,79	0,92
x19	46	22,00	61,7	64	2,80	0,96
x20	47	22,20	57,6	61	2,59	0,94
x21	48	22,50	60,7	57	2,70	1,06
x22	49	22,00	57,6	58	2,62	0,99
x23	50	21,40	58,5	59	2,73	0,99
x24	51	20,00	60,5	62,5	3,03	0,97
x25	52	19,90	61,5	61,8	3,09	1,00
x26	53	20,00	61,3	63	3,07	0,97
x27	54	18,40	61,4	62,6	3,34	0,98
x28	55	18,20	61,3	61,5	3,37	1,00
x29	56	19,00	NA	62	-	-
x30	57	18,20	55	64	3,02	0,86
x31	58	18,00	56	62	3,11	0,90
x32	59	18,00	55,6	61,5	3,09	0,90
x33	60	17,90	52	62	2,91	0,84
x34	61	17,00	52,4	61,3	3,08	0,85

x35	62	17,30	NA	59,4	-	-
x36	63	16,60	50	59	3,01	0,85
x37	64	16,00	56	60,3	3,50	0,93
x38	65	16,00	54	59	3,38	0,92
x39	66	17,70	52,4	57,3	2,96	0,91
x40	67	17,00	48,9	55	2,88	0,89
x41	68	15,60	47,3	52,7	3,03	0,90
x42	69	15,00	44,8	51	2,99	0,88
x43	70	14,70	NA	46,9	-	-
x44	71	14,00	39,2	45,7	2,80	0,86
x45	72	12,50	35,6	44,7	2,85	0,80
x46	73	13,10	33,2	38,4	2,53	0,86
x47	74	12,10	29,7	36,1	2,45	0,82
x48	75	10,70	27,9	31,7	2,61	0,88
x49	76	10,20	24,9	27,3	2,44	0,91
x50	77	9,50	22,4	23,2	2,36	0,97
x51	78	8,90	20,4	19,7	2,29	1,04
x52	79	8,97	21,1	17,2	2,35	1,23
x53	80	8,90	16	11,40	1,80	1,40

Table S2 continued. Measurements (mm) for selected vertebral centra of ***Arthropterygius chrisorum*** SGM 1573.

**Centrum position**	**L**	**H**	**W**	**W neural canal**
At-ax	52	94	78	26
v3. ant. presacr.	32	82	77	NA
ant. presacr.	43	98	105	30
post. presacr.	46	112	117	28
post. presacr.	46	112	121	24

Table S3. Selected measurements (mm) for ***Arthropterygius lundi***

**Scapula**	**SGM 1731-01–15**	**SGM 1502**	
Width of proximal end	135	134[as preserved]	
Thickness of proximal end	43	42	
Width of the shaft (aprox. midlength)	62	63	
Maximal tickness of the shaft	21	24	
**Coracoid**	**SGM 1731-01–15**	**PMO 222.654**	
Anteroposterior length	235	219	
Mediolateral width	190	213	
Length of medial symphysis	133	156	
Thickness of the medial facet	50	52	
Length of glenoid contribution	106	80	
Length of scapular facet	47	45	
**Humerus**	**SGM 1731-01–15**	**SGM 1502**	**PMO 222.654**
Proximodistal length	135	-	152
Width of proximal end	109	-	104
Height (thickness) of proximal end	57[compressed]	-	65
Width of distal end (fU/fR/faae)	127[rec.] (?/46/36)	145(69/58/37)	135(61/49/25)
Thickness of distal end (fU/fR/faae)	45(32/45/34)	52 (41/52/38)	50(40/50/35)
Width of the shaft	85	100	85
**Selected anterior presacral centrum**	**SGM 1731-01–15**	**SGM 1502**	**PMO 222.654**
Length	32	35	30
Width	67	73	65
Height	65	80	70

Table S4. Selected measurements (mm) for ***Arthropterygius volgensis*** KSU 982/P-213

**Basisphenoid**	
Length	52
Width (max/bpt)	65/50
Height	27
**Quadrate** (measurements are identical for left and right elements)	
Maximal length of dorsal portion (region of occipital lamella)	40
Maximal length of ventral portion (condylar region)	50
Width of the condyle (acond)	30
Height	74
Length in the region of quadrate foramen	35
**Opisthotic** (measurements are identical for left and right elements)	
Mediolateral length	40
Width of the medial head	25
Height of the medial head	30
**Articular**	
Length	35
Height	30
**Coracoid** (measurements are identical for left and right elements)	
Anteroposterior length	130
Mediolateral width	115
Length of medial symphysis	90
Thickness of the medial facet	30
Length of glenoid contribution	55
Length of scapular facet	20

Institutional abbreviations for tables S5-S8

CAMSM, Sedgwick Museum of Earth Sciences, Cambridge, UK; **CCMGE,** Chernyshev's Central Museum of Geological Exploration, Saint Petersburg, Russia; **CMN,** Canadian Museum of Nature, Ottawa, Canada; **GLAHM**, The Hunterian Museum, University of Glasgow, Glasgow, UK; **KSU,** A.A. Shtukenberg Museum of Geology and Mineralogy of Kazan State University, Kazan, Russia; **NHMUK,** Natural History Museum, London, UK; **OUMNH,** Oxford University Museum of Natural History, UK; **PIN,** Borissiak Paleontological Institute of the Russian Academy of Sciences, Moscow, Russia; **PMO,** Natural History Museum, University of Oslo (Palaeontological collection), Oslo, Norway; **QM**, Queensland Museum, Brisbane, Australia; **RBINS**, Royal Belgian Institute of Natural Sciences, Brussels, Belgium; **RGHP,** Réserve naturelle Géologique de Haute-Provence, Digne-les-bains, France; **SGM,** V.I. Vernadsky State Geological Museum of the Russian Academy of Sciences, Moscow, Russia; **SMSS**, Städtisches Museum Schloss Salder, Salzgitter, Germany; **SRM**, Syzran Regional Museum, Syzran, Russia; **UPM,** Undory Palaeontological museum, Undory, Ulyanovsk Region, Russia; **UW,** University of Wyoming, Laramie,Wyoming, USA; **WESTM,** Weston-super-Mare Museum, Weston-super-Mare, UK; **YKM,** I.A. Goncharov Ulyanovsk Regional Museum, Ulyanovsk, Russia.

Table S5. Basisphenoid proportions in ophthalmosaurids

Taxon (specimen)	Max width of bsph. at anterior bpt.	Maximal length of basisph.	Width to length ratio	Source
Arthropterygius chrisorum (CMN 40608)	**98**	**78**	**1.256**	Maxwell 2010; NGZ pers. obs. on photographs
Arthropterygius cf. *A. chrisorum* (SGM 1743-1)	**86**	**73**	**1.178**	NGZ pers. obs.
A. chrisorum (CCMGE 17-44/13328)	**74**[est]	**64**	**1.156**	NGZ pers. obs.
A. chrisorum (CCMGE 3-16/13328)	**68**[est]	**57**	**1.192**	NGZ pers. obs.
A. chrisorum (PMO 222.669)	**83**	**73**	**1.136**	NGZ pers. obs.
A. lundi (SGM 1502)	**102**	**80**	**1.275**	Zverkov *et al*. 2015a; NGZ pers. obs.
A. volgensis (KSU 982/P-213)	**50**	**52**	**0.961**	NGZ pers. obs.
A. hoybergeti (SVB 1451)	**90**[est]	**80**	**1.125**	NGZ pers. obs.
Ophthalmosaurus icenicus (NHMUK R. 2149)	**105**	**72**	**1.458**	Andrews 1910
O.icenicus (NHMUK R. 2162)	**119**	**75**	**1.587**	
O.icenicus (NHMUK R. 2740)	**110**	**87**	**1.264**	
O.icenicus (NHMUK R. 2161)	**92**	**64**	**1.438**	
O.icenicus (NHMUK R. 2155)	**98**	**76**	**1.289**	
Acamptonectes densus (GLAHM 132588)	est.130	95	1.368	Fischer *et al*. 2012
Platypterygius australis (QM F116940)	115	90	**1.278**	Kear 2005
in utero P. australis (QM F16812)	39	32	**1.219**	Kear & Zammit 2014
P. platydactylus (lost type specimen)	**110**	**85**	**1.294**	Broili 1907
Sisteronia seeylei (RGHP SI 2)	90	75	**1.200**	Fischer *et al*. 2014
Sveltonectes insolitus (RBINS R269)	54	45	**1.200**	Fischer *et al*. 2011; NGZ pers. obs.
Plutoniosaurus bedengensis (UPM 2/740)	**104**	**80**	**1.300**	NGZ pers. obs.
U. gorodischensis (UPM EP-II-20(572))	**134**	**80**	**1.675**	Zverkov & Efimov 2018; NGZ pers. obs.
U. nessovi (UPM EP-II-24(785))	**138**	**80**	**1.725**	Zverkov & Efimov 2018; NGZ pers. obs.
Grendelius mordax (CAMSM J68516)	**113**	**75**	**1.507**	McGowan, 1976; NGZ pers. obs.
G. alekseevi (YKM 56702)	**131**	**89**	**1.472**	Zverkov *et al*. 2015b; NGZ pers. obs.
G. pseudoscythicus (UPM 3/100)	**105**	**65**	**1.615**	NGZ pers. obs.

*measurements from the photographs are in regular font, direct measurements are in bold font.

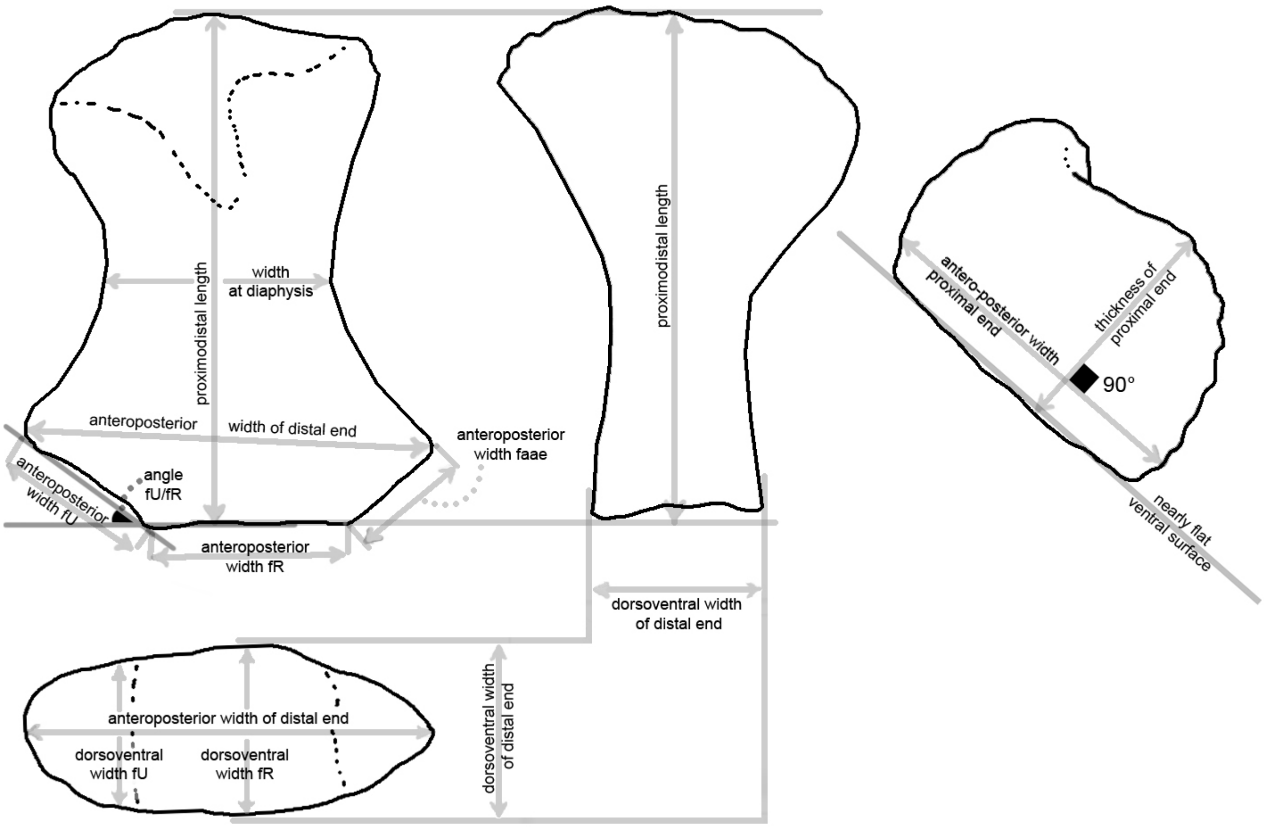

Figure S1. Scheme of measurements of the humerus applied herein. More common is metric "dorsoventral width" of proximal end of the humerus, however, considering an existing torsion between the proximal and distal ends of the humerus, precise identification of what is "dorsoventral width" became problematic especially in humeri with markedly anteroposteriorly elongated proximal ends and low angles of humeral torsion. By this reason, here we use another similar metric "thickness of proximal end" which could be defined as maximal height of the humeral proximal end measured perpendicular to the flat ventral surface of the humerus.

Table S6. Measurements of ophthalmosaurid humeri (mm). In grey cells are specimens for which measurement are taken from the literature.

Taxon (specimen)	L / R	Proximo-distal length	Antero-posterior width proximal end	Thick-ness of proximal end	Antero-posterior width of distal end	Dorso-ventral width of distal end	Width at diaphysis	Anteroposterior width			Dorso-ventral width		Angle between fR and fU in dorsal view (°)
								fU	fR	faae	fU	fR	
***Arthropterygius chrisorum* (CMN 40608)**	L	220	172	100	195	*c*.85	140	70	75	65	*c*.60	*c*.85	35
	R	213	171	?	196	?	?	?	?	?	?	?	?
***A. chrisorum* (SGM 1573)**	L	220	185	95	?	80	125	?	68	58	*c*.51	76	?
***A. chrisorum* (CCMGE 17-44/13328)**	R	151	121	60	122	47	84	50	45	39	35	47	32
***A. chrisorum* (CCMGE 3-16/13328)**	R	99	73	27	77,3	30	60	37	36	25	22	30	45
***A. chrisorum* (PMO 222.669)**	L	160	124	62	121	54	77	52	49	40	41	54	35
	R	163	113.5	63	120	50	78	52	51	37	39.3	48.7	35
***A.* cf. *chrisorum* (PMO 224.250)**	L	190	133	87	146	55.5	93	57	56	47	48	55	40
	R	190	139	89.5	154	60	96.6	63	61	50	52	60	40
***Arthropterygius hoybergeti* (YKM 63548)**	L	170	131	80	139	60	100	65	64	24	44	55	45
	R	167	127	85	132	62	90	62	60	24	50	62	38
***Arthropterygius hoybergeti* (UPM 2442)**	L	163	121	68	110	60	80	55	50	20	47	60	35
	L	152	110	68	136	50	85	61	48	30	39	50	30

Arthropterygius lundi **(PMO222.654)**	R	155	105	60	130	50	85	60	50	30	40	50	33
Brachypterygius extremus **(NHMUK R3177)**	R	150	92	NA	108	48	*c.*60	44	50	NA	38	30	65
B. extremus **(WESTM 78/219)**	L	147	92	*c.*40	112	50	*c.*65	44	51	NA	39	30	60
B. extremus **(CAMSM 29803)**	L	120	81	42	80	42	47	35	40	NA	42	42	60
B. extremus **(CAMSM 67556)**	L	124	85	49	95	45	54	40	40	NA	45 est.	45	60
Grendelius alekseevi **(YKM 56702)**	L	154	104	55	106	40	85	40	47	NA	38	38	75
	R	155	106	55	109	40	83	42	44	NA	38	38	65
G. zhuravlevi **(SRM Hb 30192)**	L	124	98	38	93	50	80	40	37	NA	50	47	60
G. zhuravlevi **(PIN 426/60-76)**	L	144	119	46	87	45	70	40	40	NA	45	43	77
Ophthalmosaurus icenicus **(NHMUK R. 2134)**	L	170	130	87	145	55	80	49	63	34	50	55	40
O. icenicus **(CAMSM X 50333)**	L	122	77	55	113	46	75	46	48	25	42	46	40
O. icenicus **(CAMSM X 50329)**	L	61	45	26	55	23	35	25	24	12	22	23	45
O. icenicus **(CAMSM X 50327)**	R	175	110	83	140	60	87	68	60	32	60	58	45
O. icenicus **(CAMSM X 50326)**	L	168	110	68	143	54	85	60	68	28	45	54	38
O. icenicus **(CAMSM J67367)**	L	155	110	70	130	55	85	50	55	30	45	55	35
O. icenicus **(CAMSM J67368)**	R	160	115	70	130	55	85	50	55	30	45	55	35
O. icenicus **(CAMSM J65930)**	R	182	120	80	172	62	90	62	80	33	58	62	37
O. icenicus **(CAMSM J66012)**	L	170	115	80	167	65	87	60	65	45	53	65	35
O. icenicus **(CAMSM J66013)**	R	143	88	50	115	53	73	50	58	30	40	50	45
O. icenicus **(CAMSM J65451)**	L	145	113	68	132	55	85	50	60	35	42	55	40
O. icenicus **(CAMSM J65452)**	L	155	108	72	140	58	70	49	55	40	48	58	37
O. icenicus **(CAMSM J65713)**	R	140	110	75	120	48	64	45	53	30	40	48	40
O. icenicus **(CAMSM J65089)**	L	140	88	58	128	50	71	50	60	30	40	50	45
O. icenicus **(CAMSM J65196)**	R	145	95	72	142	50	82	60	62	32	40	50	40
O. icenicus **(SKM OF 242/1-19)**	R	200	136	93	140	60	86	50	65	35	60	58	45
Platypterygius americanus **(UW 2421)**	R	140	95	*c.*70	107	*c.*55	70 est.	56	47	NA	?	?	40
	L	135	82est.	?	104	?	66 est.	?	?	NA	?	?	?
Platypterygius americanus **(UW 5547)**	L	103	NA	NA	68	38	33	36	32	NA	38	36	60
	R	100	62 [?incompl.]	35	70	33	47	38	35	NA	33	31	65

P. australis **(QM F 2573)**	R	170	113	82	143	58	90	65	55	23	58	58	35
P. hercynicus **(SMSS "SGS")**	R	122	90	47	99	52	60	40	35	0.7	44	52	25
	L	109	100	55	102	49	72	40	37	11	41	49	20
P. platydactylus **(Broili 1907)**	L	125	65	60 *ca.*	80	43	*c*.50	40	36	0.5	39	43	40
Plutoniosaurus bedengensis **(UPM 2/740)**	L	130	105	62	88	47	55	44	38	10	47	47	30
	R	125	107	54	90	45	55	43	40	10	45	45	30
Sveltonectes insolitus **(RBINS R269)**	L	88	78	50	60	35	44	32	33	0	34	35	45
	R	90	72	52	60	34	45	32	30	0	34	34	47
Undorosaurus gorodischensis **(UPM EP-20(572))**	L	152	100	65	98	*c*.55	75	50	55	0.5	?	?	35
U. gorodischensis **(UPM EP-23(744))**	R	173	110	70	110	56	80	50	57	20	50	56	45
U. gorodischensis **UPM EP-II-20(1075)**	L	160	103	70	110	60	70	50	60	20	52	60	45
U. gorodischensis **UPM EP-II-27(870)**	L	160	120	65	110	60	74	50	53	20	51	60	40
	R	160	125	70	110	60	74	50	57	17	56	60	45
U. gorodischensis **PMO 214.578**	L	155	115	65	111	57	75	57	55	0.5	50	57	35
	R	150	120	67	115	63	73	55	50	20	57	63	40
U. nessovi **(UPM EP-II-24(785))**	R	183	107	90	135	64	77	60	65	20	55	64	40
U. trautscholdi **(SGM 1503)**	L	149	95	75	105	58	61	55	58	22	47	58	50

List of ratios in Table S7.
(1) Humeral proximal expansion: anteroposterior width of humeral proximal end divided by the humeral proximodistal length.
(2) Humeral distal expansion: anteroposterior width of humeral distal end divided by the humeral proximodistal length.
(3) Humeral stoutness: humeral minimal anteroposterior width at diaphysis divided by the humeral proximodistal length.
(4) Humeral proximodistal proportionality: anteroposterior width of humeral proximal end divided by the same measurement of its distal end.
(5) Isometry of humeral proximal end (or 'anteroposterior elongation' of humeral proximal end): anteroposterior width of humeral proximal end divided by the thickness of humeral proximal end (see Fig. S1).
(6) Humeral distal compression: anteroposterior width of humeral distal end relative to the maximal dorsoventral width of humeral distal end.
(7) Relative anteroposterior width of facet for preaxial accessory epipodial element and radial facet.
(8) Relative anteroposterior width of ulnar and radial facets.
(9) Relative dorsoventral width of ulnar and radial facets.
(10) Angle between the ulnar and radial facets (rad).

Table S7. Ratios of ophthalmosaurid humeri.

Taxon (specimen)	L/R	(1)	(2)	(3)	(4)	(5)	(6)	(7)	(8)	(9)	(10)
***Arthropterygius chrisorum* (CMN 40608)**	L	0.782	0.886	0.636	0.882	1.720	2.294	0.867	0.933	0.706	0,611
***A. chrisorum* (SGM 1573)**	L	0.841	NA	0.568	NA	1.947	NA	0.853	NA	0.670	NA
***A. chrisorum* (CCMGE17-44/13328**	R	0.801	0.808	0.556	0.992	2.017	2.596	0.867	1.111	0.671	0,559
***A. chrisorum* (CCMGE 3-16/13328)**	R	0.737	0.781	0.606	0.944	2.704	2.577	0.694	1.028	0.745	0,785
***A. chrisorum* (PMO 222.669)**	L	0.775	0.756	0.481	1.025	2.000	2.241	0.816	1.061	0.733	0,611
	R	0.696	0.736	0.479	0.946	1.802	2.400	0.725	1.020	0.759	0,611
***A.* cf. *chrisorum* (PMO 224.250)**	L	0.700	0.768	0.489	0.911	1.529	2.631	0.839	1.018	0.807	0,785
	R	0.732	0.811	0.508	0.903	1.553	2.567	0.820	1.033	0.873	0,785
***Arthropterygius hoybergeti* (YKM 63548)**	L	0.771	0.818	0.588	0.942	1.638	2.317	0.375	1.016	0.867	0,785
	R	0.760	0.790	0.539	0.962	1.494	2.129	0.400	1.033	0.800	0,663
***A. hoybergeti* (UPM 2442)**	L	0.742	0.675	0.491	1.100	1.779	1.833	0.400	1.100	0.806	0,611
***Arthropterygius lundi* (PMO222.654)**	L	0.724	0.895	0.559	0.809	1.618	2.720	0.625	1.271	0.783	0,524
	R	0.677	0.839	0.548	0.808	1.750	2.600	0.600	1.200	0.780	0,576
***Brachypterygius extremus* (NHMUK R3177)**	R	0.613	0.720	0.400	0.852	↓2.300	2.250	0.000	0.880	0.800	1,134
***B. extremus* (WESTM 78/219)**	L	0.626	0.762	0.442	0.821	c.2.300	2.240	0.000	0.863	1.267	1,047
***B. extremus* (CAMSM 29803)**	L	0.675	0.667	0.392	1.013	1.929	1.905	0.000	0.875	1.300	1,047
***B. extremus* (CAMSM 67556)**	L	0.685	0.766	0.435	0.895	1.735	2.111	0.000	1.000	1.000	1,047
***Grendelius alekseevi* (YKM 56702)**	L	0.675	0.688	0.552	0.981	1.891	2.650	0.000	0.851	1.000	1,309
	R	0.684	0.703	0.535	0.972	1.927	2.725	0.000	0.955	1.000	1,134
***G. zhuravlevi* (SRM Hb 30192)**	L	0.790	0.750	0.645	1.054	2.579	1.860	0.000	1.081	1.000	1,047
***G. zhuravlevi* (PIN 426/60-76)**	L	0.826	0.604	0.486	1.368	2.587	1.933	0.000	1.000	1.064	1,344
***Ophthalmosaurus icenicus* (NHMUK R. 2134)**	L	0.765	0.853	0.471	0.897	1.494	2.636	0.540	0.778	1.047	0,698
**CAMSM X 50033**	L	0.631	0.926	0.615	0.681	1.400	2.457	0.521	0.958	0.909	0,698
**CAMSM X 50329**	L	0.738	0.902	0.574	0.818	1.731	2.391	0.500	1.042	0.913	0,785
**CAMSM X 50327**	R	0.629	0.800	0.497	0.786	1.325	2.333	0.533	1.133	0.957	0,785
**CAMSM X 50326**	L	0.655	0.851	0.506	0.769	1.618	2.648	0.412	0.882	1.034	0,663
**CAMSM J67367**	L	0.710	0.839	0.548	0.846	1.571	2.364	0.545	0.909	0.833	0,611
**CAMSM J67368**	R	0.719	0.813	0.531	0.885	1.643	2.364	0.545	0.909	0.818	0,611
**CAMSM J65930**	R	0.659	0.945	0.495	0.698	1.500	2.774	0.413	0.775	0.818	0,646
**CAMSM J66012**	L	0.676	0.982	0.512	0.689	1.438	2.569	0.692	0.923	0.935	0,611
**CAMSM J66013**	R	0.615	0.804	0.510	0.765	1.760	2.170	0.517	0.862	0.815	0,785
**CAMSM J65451**	L	0.779	0.910	0.586	0.856	1.662	2.400	0.583	0.833	0.800	0,698
**CAMSM J65452**	L	0.697	0.903	0.452	0.771	1.500	2.414	0.727	0.891	0.764	0,646
**CAMSM J65713**	R	0.786	0.857	0.457	0.917	1.467	2.500	0.566	0.849	0.828	0,698
**CAMSM J65089**	L	0.629	0.914	0.507	0.688	1.517	2.560	0.500	0.833	0.833	0,785
**CAMSM J65196**	R	0.655	0.979	0.566	0.669	1.319	2.840	0.516	0.968	0.800	0,698
***O. icenicus* (SKM OF 242/1-19)**	R	0.680	0.700	0.430	0.971	1.462	2.333	0.538	0.769	0.800	0,785
***Platypterygius americanus* (UW 2421)**	R	0.679	0.764	0.500	0.888	1.357	1.945	0.000	1.191	1.034	0,698
***P. americanus* (UW 5547)**	R	0.620	0.700	0.470	0.886	1.771	2.121	0.000	1.086	1.056	1,047
***P. australis* (QM F 2573)**	R	0.665	0.841	0.529	0.790	1.378	2.466	0.418	1.182	1.056	0,611
***P. hercynicus* (SMSS "SGS")**	R	0.738	0.811	0.492	0.909	1.915	1.904	0.020	1.143	1.000	0,436
	L	0.917	0.936	0.661	0.980	1.818	2.082	0.297	1.081	0.846	0,349
***P. platydactylus* (Broili 1907)**	L	0.520	0.640	0.400	0.813	1.083	1.860	0.014	1.111	0.837	0,698
	L	0.808	0.677	0.423	1.193	1.694	1.872	0.263	1.158	0.907	0,524

***P. bedengensis* (UPM 2/740)**	R	0.856	0.720	0.440	1.189	1.981	2.000	0.250	1.075	1.000	0,524
***Sveltonectes insolitus* (RBINS R269)**	L	0.886	0.682	0.500	1.300	1.560	1.714	0.000	0.970	1.000	0,785
	R	0.800	0.667	0.500	1.200	1.385	1.765	0.000	1.067	0.971	0,82
***U. gorodischensi* (UPM EP-20(572))**	L	0.658	0.645	0.493	1.020	1.538	1.782	0.009	0.909	1.000	0,611
***U. gorodischensis* (UPM EP-23(744))**	R	0.636	0.636	0.462	1.000	1.571	1.964	0.351	0.877	1.000	0,785
***U. gorodischensis* (UPM EP-II-20(1075))**	L	0.644	0.688	0.438	0.936	1.471	1.833	0.333	0.833	0.893	0,785
***U. gorodischensis* (UPM EP-II-27(870))**	L	0.750	0.688	0.463	1.091	1.846	1.833	0.377	0.943	0.867	0,698
	R	0.781	0.688	0.463	1.136	1.786	1.833	0.298	0.877	0.850	0,785
***U. gorodischensis* (PMO 214.578)**	L	0.742	0.716	0.484	1.036	1.769	1.947	0.009	1.036	0.933	0,611
	R	0.800	0.767	0.487	1.043	1.791	1.825	0.400	1.100	0.877	0,698
***U. nessovi* (UPM EP-II-24(785))**	R	0.585	0.738	0.421	0.793	1.189	2.109	0.308	0.923	0.905	0,698
***U. trautscholdi* (SGM 1503)**	L	0.638	0.705	0.409	0.905	1.267	1.810	0.379	0.948	0.859	0,873
Mean value		0,713	0.780	0.503	0.927	1.699	2.236	0.371	0.985	0.901	0.750
Standard deviation		0,078	0.096	0.063	0.152	0.332	0.323	0.280	0.120	0.126	0.201

Table S8. Matrix for PCA.

Taxon (specimen)	L/R	(1)	(2)	(3)	(4)	(5)	(6)	(7)	(8)	(9)	(10)
Arthropterygius chrisorum (CMN 40608)	L	0.8823	1.1080	0.2117	-0.5918	0.0633	0.1799	1.7702	-0.4306	-1.5486	-0.6950
A. chrisorum (CCMGE 17-44/13328)	R	1.1324	0.2911	0.0846	0.1303	0.9568	1.1138	1.7702	1.0509	-1.8250	-0.9550
A. chrisorum (CCMGE 3-16/13328)	R	0.3125	0.0084	0.1636	-0.1818	3.0262	1.0547	1.1552	0.3565	-1.2406	0.1750
A. chrisorum (PMO 222.669)	L	0.7949	-0.2474	-0.0345	0.3473	0.9066	0.0147	1.5905	0.6352	-1.3307	-0.6950
	R	-0.2139	-0.4563	-0.0388	-0.1721	0.3090	0.5077	1.2660	0.2884	-1.1249	-0.6950
A. cf. *chrisorum* (PMO 224.250)	L	-0.1667	-0.1206	-0.0215	-0.4016	-0.5128	1.2218	1.6724	0.2738	-0.7462	0.1750
	R	0.2382	0.3180	0.0086	-0.4566	-0.4395	1.0237	1.6024	0.3982	-0.2244	0.1750
Arthropterygius hoybergeti (YKM 63548)	L	0.7383	0.3922	0.1353	-0.1944	-0.1852	0.2497	0.0143	0.2552	-0.2725	0.1750
	R	0.6087	0.1085	0.0570	-0.0650	-0.6171	-0.3312	0.1036	0.4028	-0.8016	-0.4350
A. hoybergeti (UPM 2442)	L	0.3760	-1.0953	-0.0194	0.8421	0.2422	-1.2466	0.1036	0.9583	-0.7504	-0.6950
Arthropterygius lundi (PMO222.654)	L	0.1370	1.1952	0.0892	-1.0735	-0.2450	1.4985	0.9071	2.3819	-0.9339	-1.1300
	R	-0.4562	0.6116	0.0720	-1.0810	0.1536	1.1269	0.8179	1.7917	-0.9603	-0.8700
Brachypterygius extremus (NHMUK R3177)	R	-1.2778	-0.6250	-0.1635	-0.7904	1.8102	0.0433	-1.3250	-0.8750	-0.8016	1.9200
B. extremus (WESTM 78/219)	L	-1.1173	-0.1885	-0.0965	-0.9906	1.8102	0.0124	-1.3250	-1.0188	2.9021	1.4850
B. extremus (CAMSM 29803)	L	-0.4872	-1.1806	-0.1767	0.2664	0.6915	-1.0255	-1.3250	-0.9167	3.1667	1.4850
B. extremus (CAMSM 67556)	L	-0.3528	-0.1445	-0.1072	-0.5083	0.1075	-0.3867	-1.3250	0.1250	0.7857	1.4850
Grendelius alekseevi (YKM 56702)	L	-0.4830	-0.9551	0.0777	0.0601	0.5780	1.2817	-1.3250	-1.1161	0.7857	2.7950
	R	-0.3734	-0.7997	0.0516	0.0031	0.6876	1.5139	-1.3250	-0.2538	0.7857	1.9200
G. zhuravlevi (SRM Hb 30192)	L	0.9913	-0.3125	0.2257	0.5379	2.6504	-1.1641	-1.3250	0.8007	0.7857	1.4850
G. zhuravlevi (PIN 426/60-76)	L	1.4537	-1.8316	-0.0268	2.6041	2.6746	-0.9370	-1.3250	0.1250	1.2923	2.9700
Ophthalmosaurus icenicus (NHMUK R. 2134)	L	0.6629	0.7598	-0.0514	-0.4964	-0.6167	1.2395	0.6024	-1.7269	1.1549	-0.2600
CAMSM X 50033	L	-1.0494	1.5232	0.1774	-1.9117	-0.9006	0.6827	0.5351	-0.2222	0.0642	-0.2600
CAMSM X 50329	L	0.3167	1.2671	0.1123	-1.0120	0.0957	0.4808	0.4607	0.4722	0.0956	0.1750
CAMSM X 50327	R	-1.0824	0.2083	-0.0093	-1.2256	-1.1256	0.3013	0.5798	1.2361	0.4406	0.1750

CAMSM X 50326	L	-0.7466	0.7416	0.0047	-1.3340	-0.2450	1.2760	0.1456	-0.8554	1.0594	-0.4350
CAMSM J67367	L	-0.0426	0.6116	0.0720	-0.8279	-0.3843	0.3952	0.6231	-0.6326	-0.5370	-0.6950
CAMSM J67368	R	0.0737	0.3385	0.0448	-0.5749	-0.1691	0.3952	0.6231	-0.6326	-0.6573	-0.6950
CAMSM J65930	R	-0.6879	1.7193	-0.0135	-1.8048	-0.5994	1.6662	0.1482	-1.7500	-0.6573	-0.5200
CAMSM J66012	L	-0.4683	2.1078	0.0139	-1.8643	-0.7877	1.0317	1.1475	-0.5160	0.2737	-0.6950
CAMSM J66013	R	-1.2515	0.2520	0.0119	-1.3604	0.1837	-0.2049	0.5223	-1.0244	-0.6795	0.1750
CAMSM J65451	L	0.8501	1.3578	0.1321	-0.7628	-0.1122	0.5077	0.7583	-1.2639	-0.8016	-0.2600
CAMSM J65452	L	-0.2080	1.2836	-0.0816	-1.3195	-0.5994	0.5504	1.2724	-0.7841	-1.0902	-0.5200
CAMSM J65713	R	0.9322	0.8036	-0.0728	-0.3640	-0.6998	0.8173	0.6966	-1.1329	-0.5826	-0.2600
CAMSM J65089	L	-1.0824	1.3988	0.0066	-1.8717	-0.5475	1.0031	0.4607	-1.2639	-0.5370	0.1750
CAMSM J65196	R	-0.7414	2.0761	0.0992	-1.9933	-1.1432	1.8700	0.5183	-0.1438	-0.8016	-0.2600
O. icenicus (SKM OF 242/1-19)	R	-0.4231	-0.8333	-0.1159	-0.0038	-0.7128	0.3013	0.5981	-1.7981	-0.8016	0.1750
Platypterygius americanus (UW 2421)	R	-0.4414	-0.1637	-0.0048	-0.5536	-1.0297	-0.8995	-1.3250	1.7207	1.0594	-0.2600
P. americanus (UW 5547)	R	-1.1923	-0.8333	-0.0524	-0.5677	0.2182	-0.3554	-1.3250	0.8393	1.2302	1.4850
P. australis (QM F 2573)	R	-0.6192	0.6373	0.0419	-1.1960	-0.9667	0.7106	0.1685	1.6402	1.2266	-0.6950
P. hercynicus (SMSS "SGS")	R	0.3167	0.3279	-0.0178	-0.4139	0.6503	-1.0283	-1.2536	1.3155	0.7857	-1.5700
	L	2.6209	1.6227	0.2501	0.0552	0.3590	-0.4779	-0.2632	0.8007	-0.4353	-2.0050
P. platydactylus (Broili 1907)	L	-2.4744	-1.4583	-0.1635	-1.0493	-1.8544	-1.1626	-1.2754	1.0509	-0.5100	-0.2600
P. bedengensis (UPM 2/740)	L	1.2140	-1.0737	-0.1269	1.4551	-0.0164	-1.1259	-0.3852	1.4408	0.0474	-1.1300
	R	1.8333	-0.6250	-0.1000	1.4269	0.8508	-0.7307	-0.4321	0.7500	0.7857	-1.1300
Sveltonectes insolitus (RBINS R269)	L	2.2226	-1.0227	-0.0048	2.1579	-0.4187	-1.6152	-1.3250	-0.1275	0.7857	0.1750
	R	1.1154	-1.1806	-0.0048	1.5000	-0.9469	-1.4591	-1.3250	0.6806	0.5590	0.3500
U. gorodischensi (UPM EP-20(572))	L	-0.7065	-1.4090	-0.0152	0.3185	-0.4835	-1.4061	-1.2925	-0.6326	0.7857	-0.6950
U. gorodischensis (UPM EP-23(744))	R	-0.9893	-1.5017	-0.0644	0.1842	-0.3843	-0.8412	-0.0719	-0.8984	0.7857	0.1750
U. gorodischensis (UPM EP-II-20(1075))	L	-0.8878	-0.9635	-0.1040	-0.2344	-0.6855	-1.2466	-0.1345	-1.2639	-0.0646	0.1750
U. gorodischensis (UPM EP-II-27(870))	L	0.4744	-0.9635	-0.0643	0.7823	0.4432	-1.2466	0.0227	-0.3467	-0.2725	-0.2600
	R	0.8750	-0.9635	-0.0643	1.0813	0.2612	-1.2466	-0.2598	-0.8984	-0.4048	0.1750
U. gorodischensis (PMO 214.578)	L	0.3710	-0.6653	-0.0304	0.4213	0.2115	-0.8936	-1.2925	0.4280	0.2566	-0.6950
	R	1.1154	-0.1389	-0.0259	0.4703	0.2772	-1.2712	0.1036	0.9583	-0.1889	-0.2600
U. nessovi (UPM EP-II-24(785))	R	-1.6449	-0.4406	-0.1305	-1.1803	-1.5365	-0.3920	-0.2261	-0.5160	0.0299	-0.2600
U. trautscholdi (SGM 1503)	L	-0.9669	-0.7844	-0.1486	-0.4424	-1.3022	-1.3178	0.0297	-0.3060	-0.3304	0.6150

Table S9. Variable loadings.

No Variable	No PC and (% variance)									
	**PC 1** (38.12)	**PC 2** (21.92)	**PC 3** (13.89)	**PC 4** (9.57)	**PC 5** (7.64)	**PC 6** (3.99)	**PC 7** (2.89)	**PC 8** (1.84)	**PC 9** (0.094)	**PC 910** (0.052)
**1**	0.13091	0.58452	0.26816	-0.03099	0.46929	0.20525	-0.22287	0.071136	-0.49498	-0.07890
**2**	-0.4554	0.031324	0.14938	0.27561	0.44168	-0.19327	-0.32921	0.30033	0.51207	-0.01151
**3**	-0.01852	0.02406	0.030094	0.035417	0.016481	-0.00695	-0.04925	0.019007	-0.08310	0.99343
**4**	0.43184	0.36403	0.086443	-0.2316	0.023683	0.31899	0.068366	-0.1524	0.69613	0.071245
**5**	0.24034	0.13466	0.65572	0.27563	-0.20663	-0.56279	0.23153	-0.067333	0.019693	-0.01457
**6**	-0.40255	-0.14916	0.40778	0.2304	-0.01583	0.48392	0.063578	-0.59816	-0.00400	-0.00655
**7**	-0.42691	0.24188	0.17711	-0.22364	-0.1945	0.22077	0.57829	0.50666	0.000245	0.012557
**8**	0.050181	0.37071	-0.31154	0.74424	-0.37912	0.21445	-0.05410	0.12601	0.011034	-0.02152
**9**	0.32058	-0.33344	-0.07110	0.36707	0.55073	0.16	0.54691	0.1332	-0.01206	0.018656
**10**	0.28512	-0.42043	0.40947	0.021912	-0.23672	0.38402	-0.37516	0.47801	-0.02485	-0.02081

Key:

**-70 – -50**	**-50 – -30**	**-30 – -10**	**-10 – 10**	**10 – 30**	**30 – 50**	**50 – 70**	**70 – 100**

PC	Eigenvalue	% variance
**1**	3.44042	38.12
**2**	1.97846	21.922
**3**	1.25354	13.889
**4**	0.863457	9.5673
**5**	0.689532	7.6401
**6**	0.359745	3.986
**7**	0.26079	2.8896
**8**	0.165982	1.8391
**9**	0.00849679	0.094146
**10**	0.00470704	0.052155

Supplemental figures

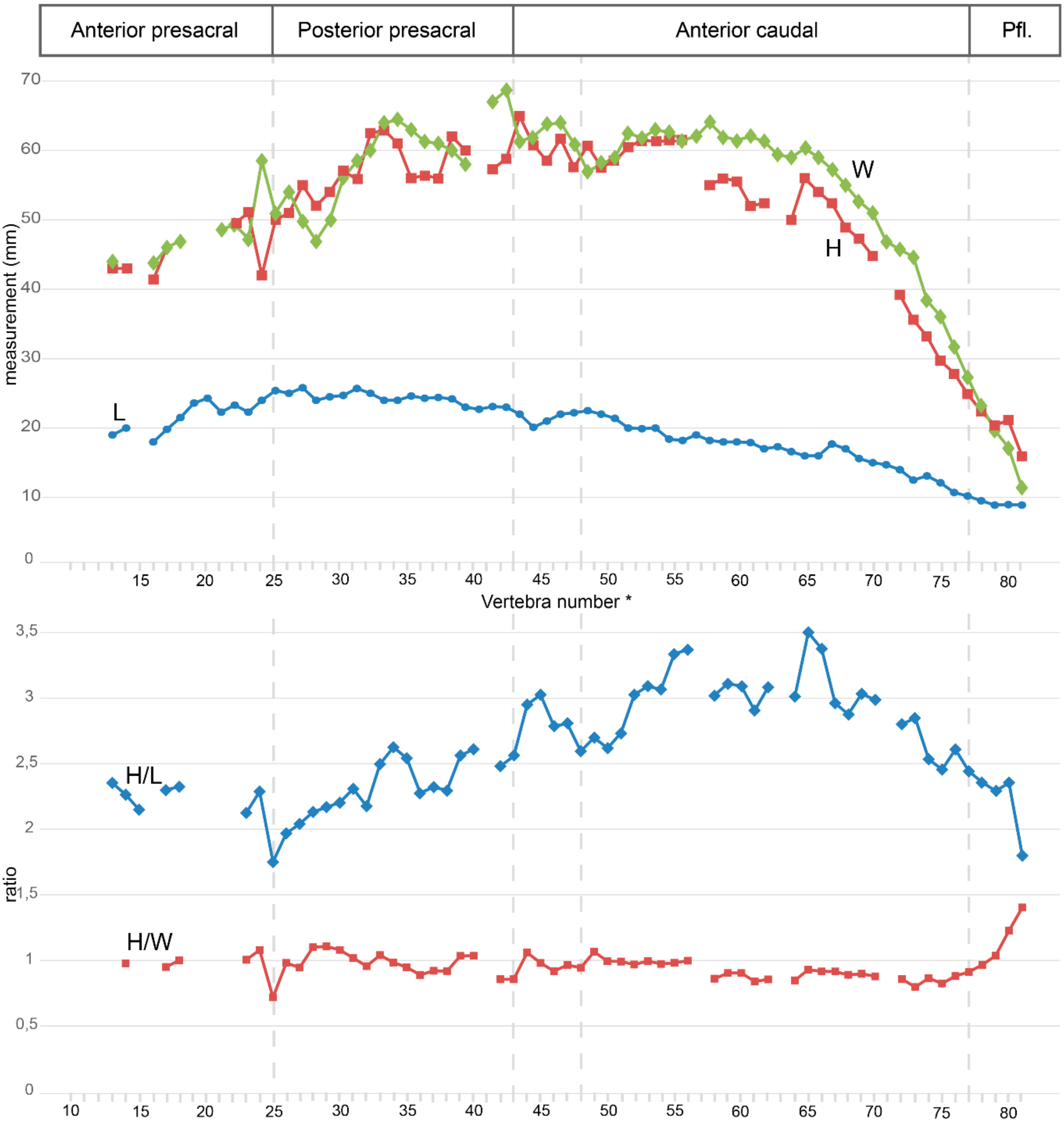

Figure S2. Vertebral dimensions of *Arthropterygius chrisorum* (CCMGE 3-16/13328). *The x-axis refers to a hypothesized position of centrum within the vertebral column: the first centrum in which diaporhysis in not fused to the neural arch was counted as 25th (as in *O. icenicus* see Moon & Kirton 2016) and the rest centra were numbered respectively).

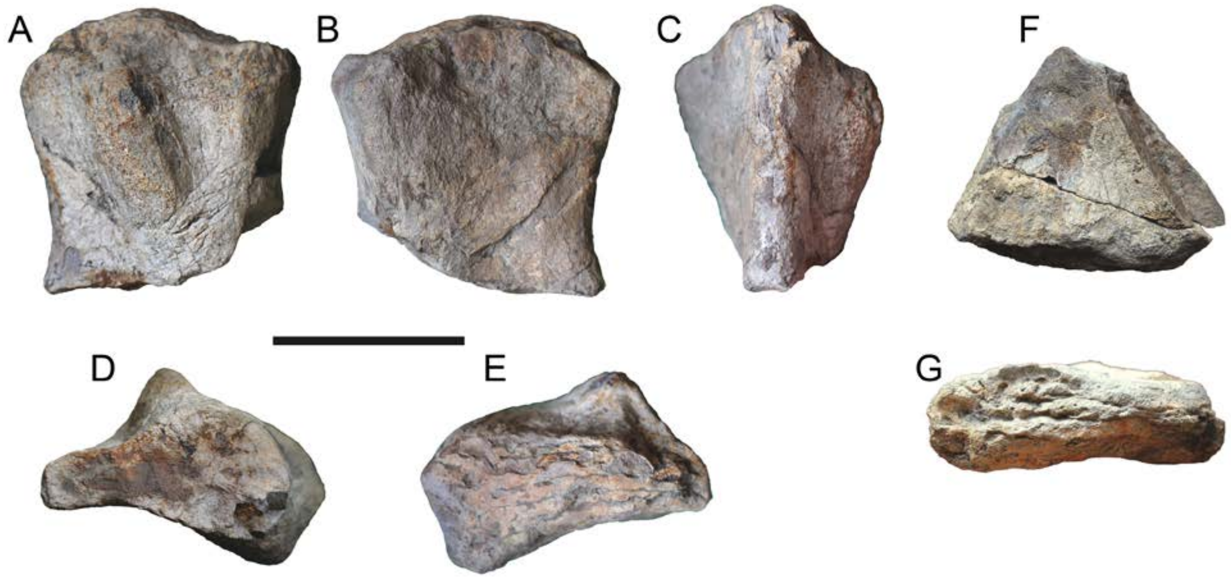

Figure S3. Proximal part of the right humerus (A–E) and glenoid portion of the ?left scapula (F–G) of Ophthalmosauridae indet. cf. *Arthropterygius* sp. CCMGE 1-2/13328. Wilczek Land. Kimmeridgian to Volgian part of the Hofer Formation. found *ex situ*. CCMGE 1-2/13328 referred to *Arthropterygius* based on strongly compressed shaft of the humerus despite over all big size and apparent maturity. A, dorsal view; B, ventral view; C, posterior view; D, cross-section of the shaft; E, proximal end of the humerus; F, dorsal view of scapular fragment; G, proximal view of scapular fragment. Scale bar represents 10 cm.

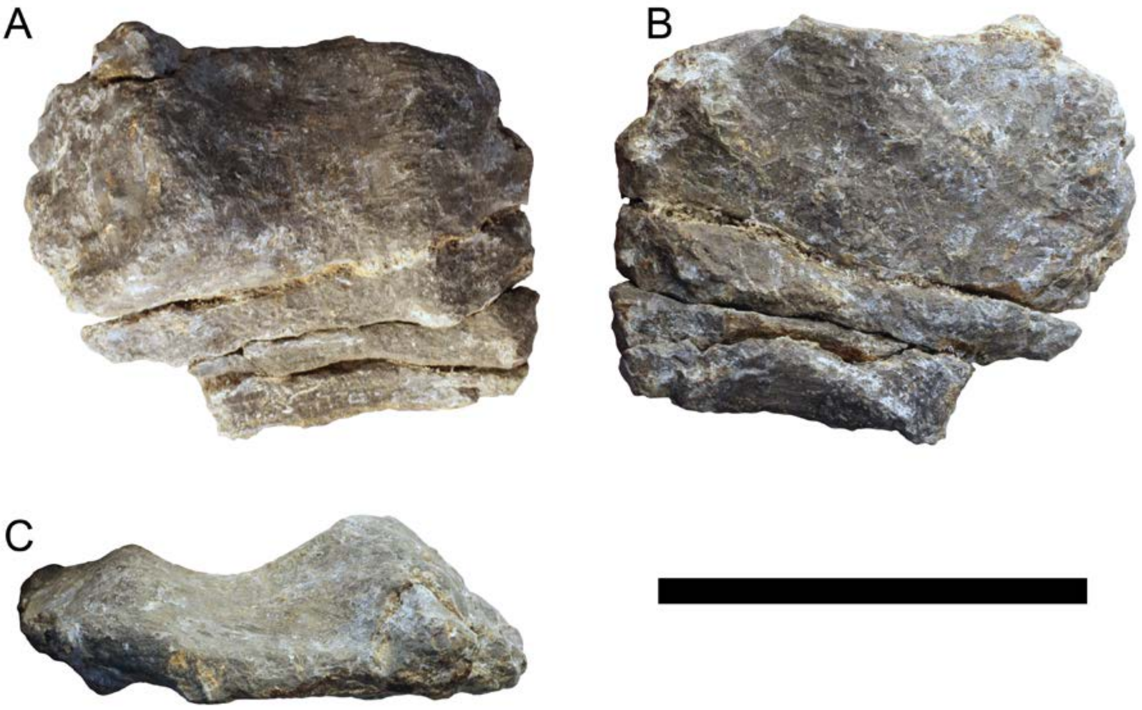

Figure S4. Left articular of *Arthropterygius hoybergeti* SVB 1451 in medial (A). lateral (B) and dorsal (C) views. Scale bar represents 5 cm.

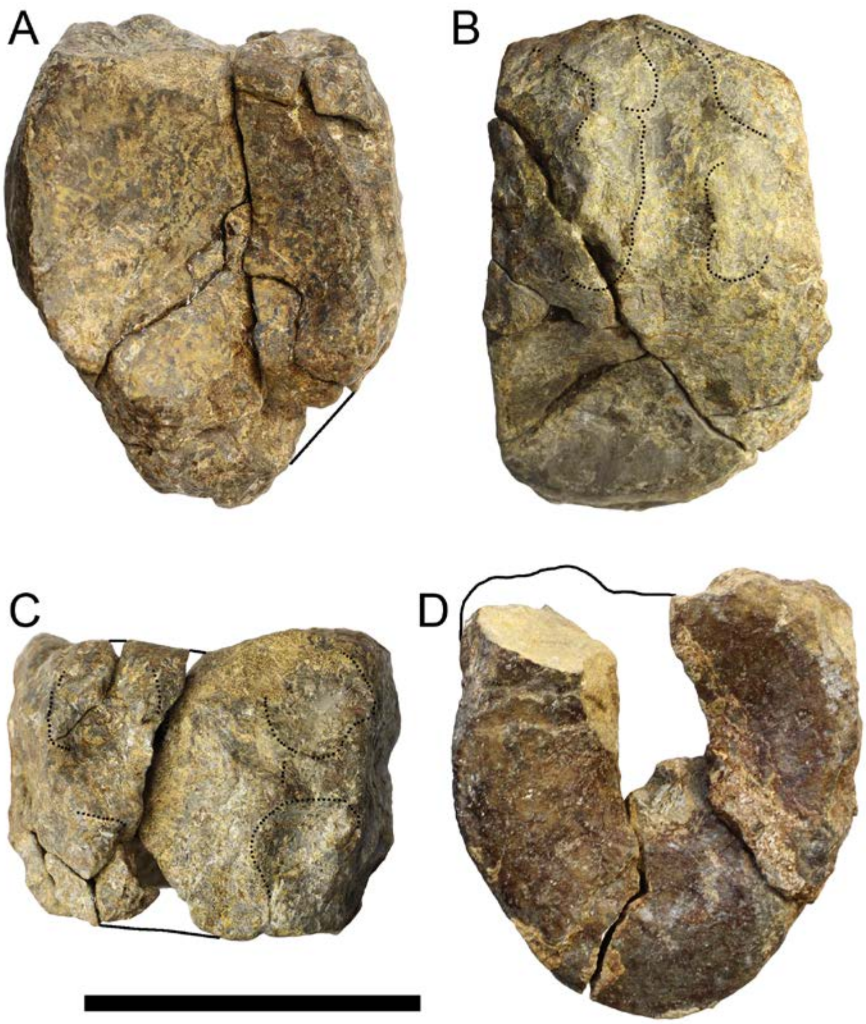

Figure S5. Atlas-axis complex and anterior presacral vertebral centrum of *Arthropterygius hoybergeti* SVB 1451. **A–C,** Atlas-axis in anterior (A), right lateral (B) and dorsal (C) views; D, articular view of presacral vertebral centrum. Scale bar represents 5 cm.

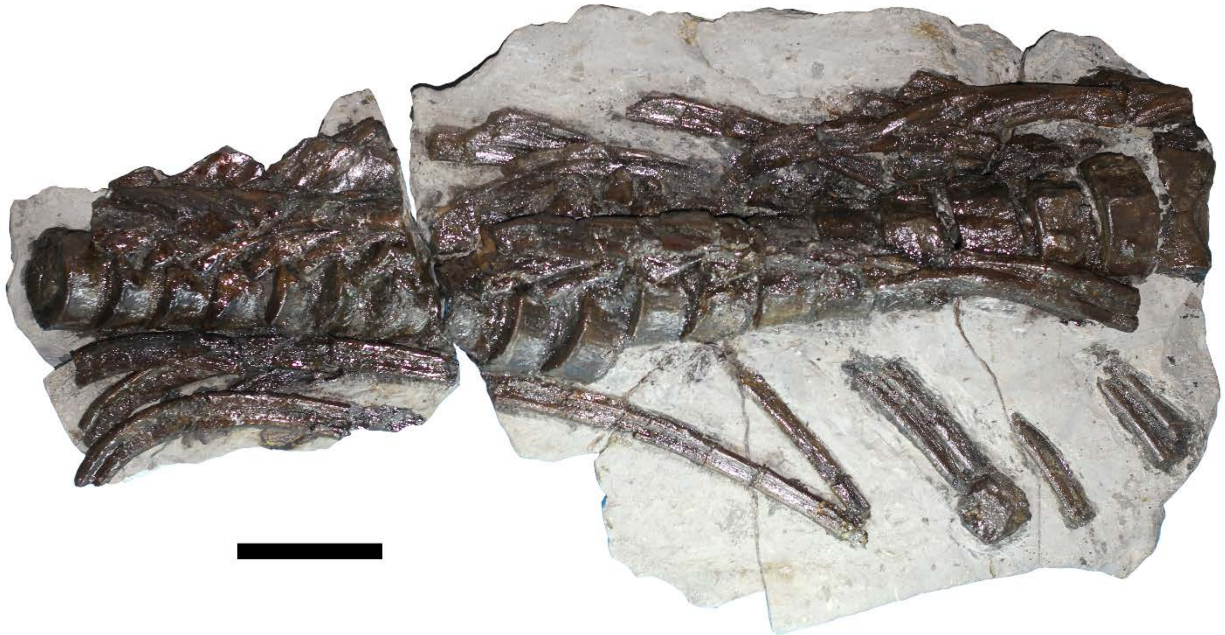

Figure S6. *Arthropterygius hoybergeti* YKM 63548. a series of presacral vertebra with articulated neural arches and ribs. Scale bar represents 10 cm.

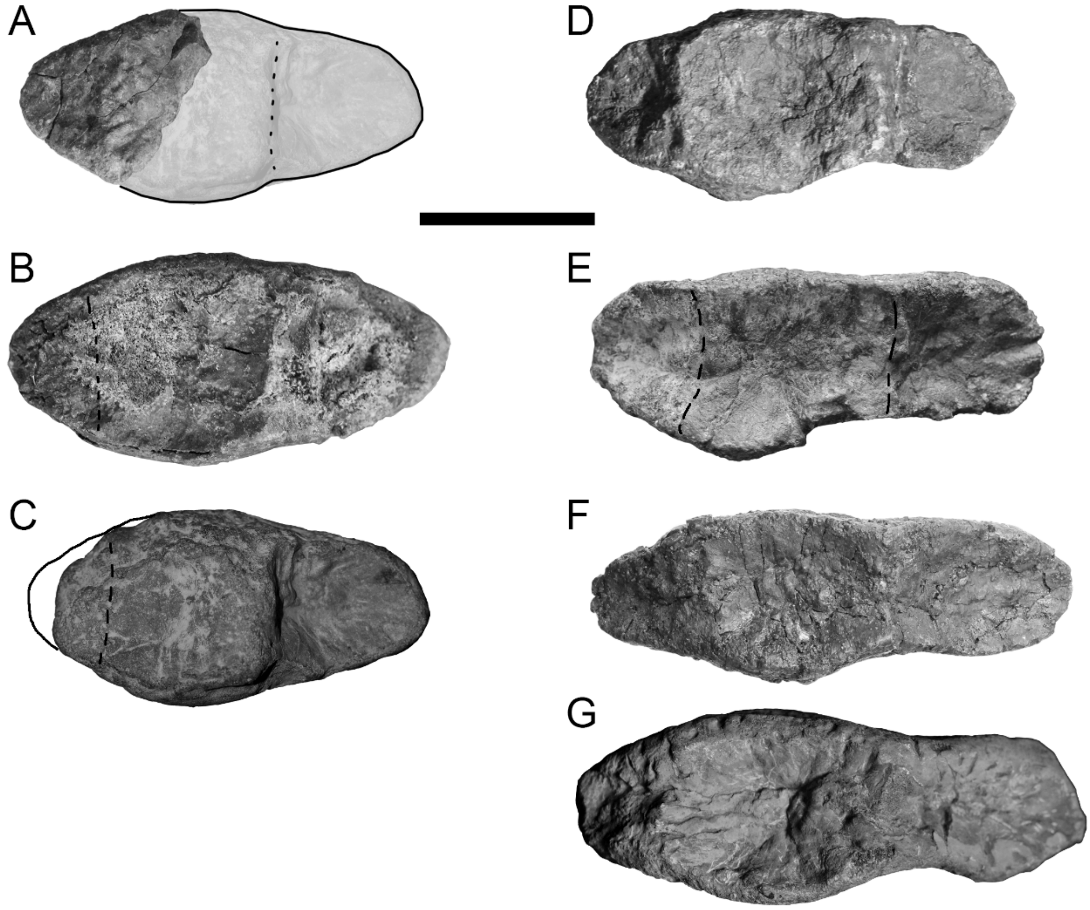

Figure S7. *Arthropterygius* distal humeral ends. **A-C,** *Arthropterygius lundi* SVB 1451 (A), YKM 63548 (B) and UPM 2442 (C); *A. chrisorum* PMO 222.669 (D) and CCMGE 17-44/13328 (E), *A. lundi* PMO 222.654 (F), SGM 1502 (G). Scale bar represents 5 cm.

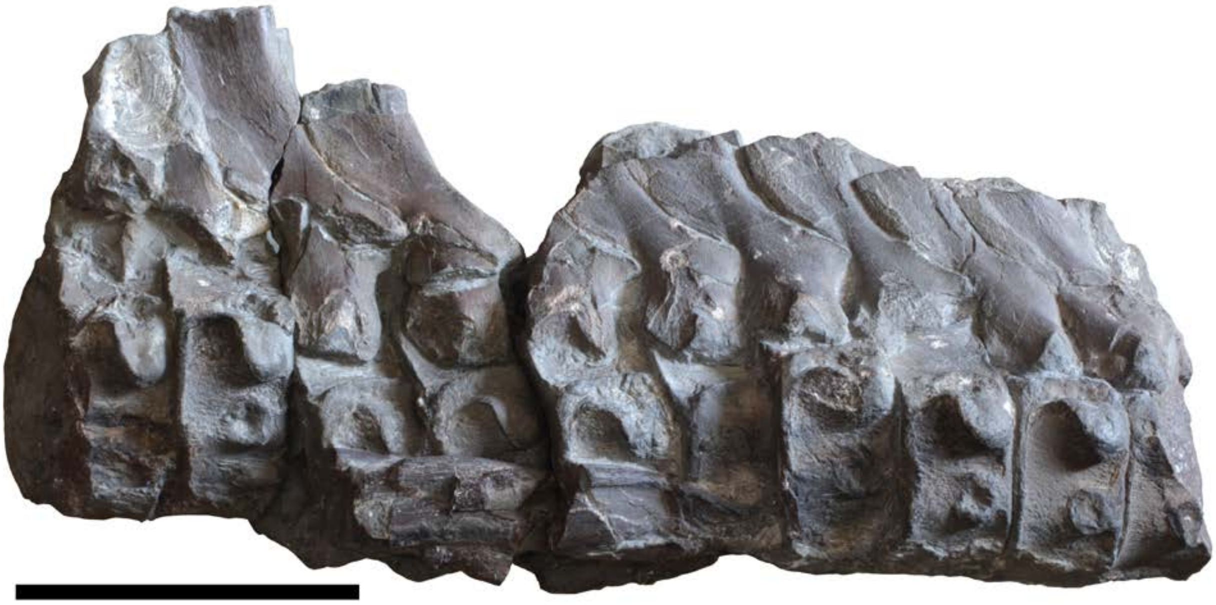

Figure S8. *Arthropterygius lundi* SGM 1731-01–15. anterior presacral vertebra with articulated neural arches. Scale bar represents 10 cm.

Modifications of character-taxon matrix

Table S10. Illustrated list of new characters		
*107	Supratemporal anteromedial tongue covering postfrontal dorsally: absent (0); present (1).	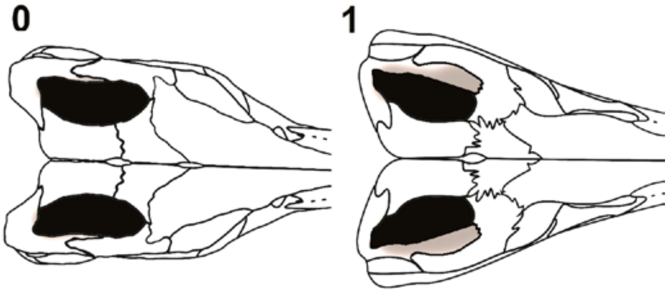
*108	Parietal medial symphysis anteroposteriorly long (0); short (1).	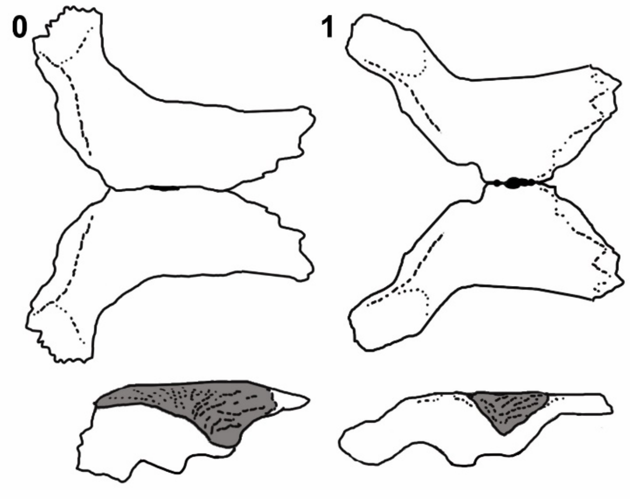
*109	Quadrate angular process present (0); extremely reduced (1)	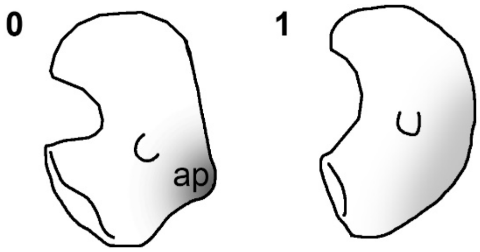
*110	Quadrate condyle massive. strongly expanded (0); weak. mediolaterally compressed* (1). *not exceeding 230 percent of quadrate mediolateral thickness in its midheight.	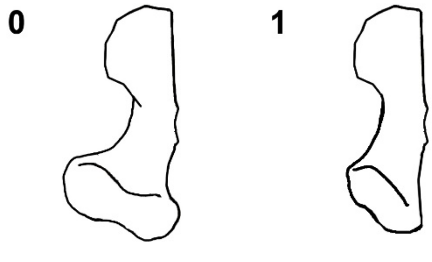
*111	Angle between the articulated coracoids: 180-160° (0). 150-130° (1). <130° (2).	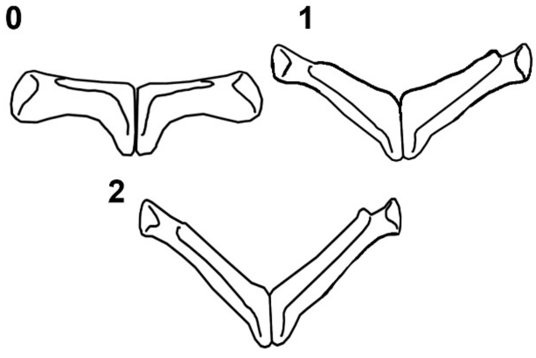

*112	Size of anterior accessory epipodial facet of the humerus: very small or even absent (0); well pronounced but not as large as radial facet (1); as large as radial facet or even larger (2).	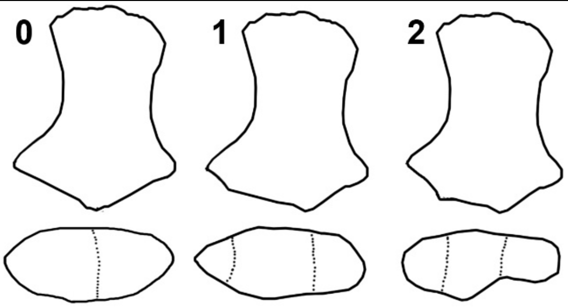

Character coding revision

The following character coding revisions were applied to the matrix of Zverkov & Efimov (2018)

Arthropterygius chrisorum: 13.?→13.0; 14.?→14.1; 16.?→16.0; 22.?→22.1; 23.?→23.0; 29.?→29.0; 31.?→31.0; 37.?→37.0; 57.?→57.0; 61.?→61.1; 65.?→65.0/1; 73.?→73.0; 76.?→76.0; 77.?→77.0; 78.?→78.1; 87.?→87.0/1; 91.?→91.1; 98.?→98.2; 99.?→99.0;

'Janusaurus' lundi: 3.?→3.0; 5.?→5.1; 21.?→21.1; 26.?→26.0; 28.1→28.0; 31.?→31.0; 32.?→32.0; 38.?→38.0; 39.?→39.0; 40.?→40.2; 58.?→58.0; 73.?→73.0; 97.0→97.?

'Palvennia' hoybergeti: 9.?→9.1; 10.0→10.1; 21.1→21.?; 28.1→28.0; 59.1→59.0; 79.?→79.1; 81.?→81.1; 83.?- 83.1; 84.?- 84.1; 87.?→87.0/1; 88.?→88.0; 89.?→89.1

(all based on new observations and conceding taxonomic revisions)

Undorosaurus gorodischensis: 28.?→28.1 (omission of Zverkov & Efimov 2018)

Paraophthalmosaurus saveljeviensis: 12.0→12.1 (new observations); 28.1→28.0 (omission of Zverkov & Efimov 2018); 41.0→41.1 (omission of Zverkov & Efimov 2018)

Sveltonectes insolitus: 49.1→49.? (new observations of NGZ - 'prootic' is in fact misidentified portion of the supraoccipital)

Character scores for *Arthropterygius volgensis*:

??????????　??????????　????????01　0????10002　1????????0　??????0???
????01??01　00?10001??　????????? ?　????????? 1　1000???101　2?

Character scores for *Arthropterygius chrisorum* PMO 222.669:

0101101?1?　?001?000??　?10000?001　0???100002　?20?1110?0　010111000?
1001???101　00010??111　12111000111 ?1010?????　?????1111　22

Table S11. Codings of new characters

Taxon	107	108	109	110	111	112
Temnodontosaurus spp.	0	0	0	0	0	0
Ichthyosaurus communis	0	0	0	0	0	0
Hauffiopteryx typicus	0	0	1	1	?	0
Stenopterygius quadriscissus	0	0	0	0	0	0
Chacaicosaurus cayi	?	?	?	?	?	0
Stenopterygius aaleniensis	0	1	?	?	0	0
Ophthalmosaurus icenicus	0	0	0	0	1	1
Ophthalmosaurus natans	0	0	?	0	1	1
Mollesaurus periallus	?	?	?	0	?	?
Acamptonectes densus	?	?	0	0	1	1
Leninia stellans	1	0	?	0	?	?
Arthropterygius chrisorum	1	?	1	1	2	2
Arthropterygius lundi	?	1	?	1	2	1
Arthropterygius hoybergeti	1	1	0	1	?	1
Grendelius alekseevi	?	?	0	0	1	0
Grendelius mordax	0	?	?	?	?	0
Caypullisaurus bonapartei	?	?	?	?	?	0
Aegirosaurus leptospondylus	0	0	?	?	?	0
Undorosaurus gorodischensis	0	0	0	0	1	1
Undorosaurus nessovi	?	?	0	0	?	1
Nannopterygius enthekiodon	?	?	?	?	0	?
Parophthalmosaurus saveljeviensis	0	1	0	0	0	1
Gengasaurus nicosiai	?	?	0	0	?	1
Athabascasaurus bitumineus	1	0	?	?	?	?
Sveltonectes insolitus	?	1	0	0	0	0
Plutoniosaurus bedengensis	?	0	0	0	0	1
Maiaspondylus lindoei	?	?	?	?	?	2
Platypterygius australis	0	0	0	0	0	1
Platypterygius hercynicus	?	0	0	0	0	1
Platypterygius americanus	0	?	?	?	?	0
Platypterygius platydactylus	?	?	0	0	?	0/1
Sisteronia seeleyi	?	?	0	0	?	1
